# 2799

**Published:** 1870-02

**Authors:** 


### 2799

Men failed in business in the United States during 1869, mainly merchants, with the honorable feelings which belong to merchants generally. The struggles they made to meet their engagements; the sacrifices they submitted to to avoid protests; the nights of sleeplessness and agony they endured in devising expedients to prevent the dreaded results; and not less terrible than any of these, the killing effort to maintain a demeanor toward wife and children which would prevent them from reading what was passing within; hoping against hope that the cloud would disperse; tortures such as these can never be estimated. And yet, every year on an average, repeats the same old story; for 1868 gave two thousand six hundred and eight failures, involving a loss of sixty-four millions of dollars. The number may be diminished for 1870, and the reader can do it for himself at least, with very considerable certainty, and thus escape the mad-house and an old age of penury and sickness, by doing two things:—

Put all your energy into your legitimate business.

Live within your income, even if you have to dwell in a shanty and live on a crust of bread.

"Why!" a man said to me one day, "I came to New York a young man. I hired a shed to carry on my business; I followed it ten years before I got able to give more than twelve and a half cents for a dinner."

He died the other day, and I asked his neighbor how much did Colonel T. leave.

"A million and a half of dollars!"

